# Gram-negative and -positive bacteria differentiation in blood culture samples by headspace volatile compound analysis

**DOI:** 10.1186/s40709-016-0040-0

**Published:** 2016-03-12

**Authors:** Michael E. Dolch, Silke Janitza, Anne-Laure Boulesteix, Carola Graßmann-Lichtenauer, Siegfried Praun, Wolfgang Denzer, Gustav Schelling, Sören Schubert

**Affiliations:** Department of Anaesthesiology, University Hospital Munich–Campus Großhadern, Ludwig-Maximilians-Universität München, Marchioninistr. 15, 81366 Munich, Germany; Department of Medical Informatics, Biometry and Epidemiology, Ludwig-Maximilians-Universität München, Marchioninistr. 15, 81366 Munich, Germany; VF Services GmbH, Andreas-Hofer-Str. 15, Absam, Austria; Wolfden Scientific Consulting, Calle Rio Segura 26, 30600 Archena, Murcia, Spain; Max von Pettenkofer-Institut für Hygiene und Medizinische Mikrobiologie, Ludwig-Maximilians-Universität München, Pettenkoferstraße 9a, 80336 Munich, Germany

**Keywords:** Mass spectrometry, Chemical ionization, Volatile compound, Blood culture, Prediction rule, Gram identification

## Abstract

**Background:**

Identification of microorganisms in positive blood cultures still relies on standard techniques such as Gram staining followed by culturing with definite microorganism identification. Alternatively, matrix-assisted laser desorption/ionization time-of-flight mass spectrometry or the analysis of headspace volatile compound (VC) composition produced by cultures can help to differentiate between microorganisms under experimental conditions. This study assessed the efficacy of volatile compound based microorganism differentiation into Gram-negatives and -positives in unselected positive blood culture samples from patients.

**Methods:**

Headspace gas samples of positive blood culture samples were transferred to sterilized, sealed, and evacuated 20 ml glass vials and stored at −30 °C until batch analysis. Headspace gas VC content analysis was carried out via an auto sampler connected to an ion–molecule reaction mass spectrometer (IMR-MS). Measurements covered a mass range from 16 to 135 u including CO_2_, H_2_, N_2_, and O_2_. Prediction rules for microorganism identification based on VC composition were derived using a training data set and evaluated using a validation data set within a random split validation procedure.

**Results:**

One-hundred-fifty-two aerobic samples growing 27 Gram-negatives, 106 Gram-positives, and 19 fungi and 130 anaerobic samples growing 37 Gram-negatives, 91 Gram-positives, and two fungi were analysed. In anaerobic samples, ten discriminators were identified by the random forest method allowing for bacteria differentiation into Gram-negative and -positive (error rate: 16.7 % in validation data set). For aerobic samples the error rate was not better than random.

**Conclusions:**

In anaerobic blood culture samples of patients IMR-MS based headspace VC composition analysis facilitates bacteria differentiation into Gram-negative and -positive.

**Electronic supplementary material:**

The online version of this article (doi:10.1186/s40709-016-0040-0) contains supplementary material, which is available to authorized users.

## Background

The occurrence of infectious complications in critically ill patients significantly impacts patient outcome by increasing the mortality rate from 11 % in non-infected patients to 25 % in patients with infection [1]. This already high mortality rate is further increased up to 55.2 % when infectious complications progress to the development of sepsis or severe sepsis [2–4], a condition found with a prevalence of 76–300 cases per 100,000 population per year in the United States, France, and Germany [2, 5, 6]. In septic patients, about 50 % of microbial proven infections are bloodstream infections with Gram-positive bacteria [2]. Although empiric antibiotic therapy is usually initiated prior to species identification in septic patients, inappropriate antibiotic therapy is present in up to 20 % of patients suffering from Gram-positive bacteraemia [4]. Thus, fast identification of the causative organism is of highest priority as this allows for early adoption of antibiotic treatment. The first step in this process, namely the detection of microorganism growth in blood culture broth bottles, has achieved high reliability and works on a semi-automatic basis. However, subsequent procedures necessary for microorganism identification as Gram-staining, species identification by matrix-assisted laser desorption/ionization time of flight mass spectrometry (MALDI-TOF MS) or biochemical methods and antibiotic susceptibility testing require staff presence [7]. This limits microorganism identification and result communication to the clinician to standard working hours. Thus, there is an ongoing urgent search for rapid and reliable diagnostic methods allowing for the identification of the causative microorganisms. An ideal method for this purpose should be able to facilitate microorganism growth detection as well as Gram and species identification in a fully automated manner.

The analysis of blood culture broth volatile fatty acid composition by gas–liquid chromatography for the detection of anaerobic bacteraemia has gained momentum back in the early 1980s [8–10]. In contrast to the analysis of volatile fatty acids by liquid chromatography, where the solvent peak usually covers high volatility compounds, direct headspace gas chromatography (GC) allows for a more detailed investigation [11]. More recently, Julák et al. applied liquid chromatography and headspace solid phase micro extraction GC mass spectrometry for the detection of volatile fatty acids originating from anaerobes in clinical samples of blood cultures and bronchoalveolar lavage fluid [12, 13]. However, the need for sample preparation prior to analysis still represents a drawback of this method. The advent of sophisticated methods such as chemical noses, direct mass spectrometry systems like ion–molecule reaction mass spectrometry (IMR-MS) or selected ion flow tube MS (SIFT-MS) nowadays allow for direct analyses of gaseous samples [14–18]. Applying these systems to in vitro analysis of bacterial samples headspace volatile compound composition makes microorganism differentiation possible even down to the species level [14–16, 19–21]. Although these results are extremely promising it must be noted that they were obtained under tightly controlled conditions with regard to broth medium, incubation time and number of inoculated microorganisms. Consequently, the next step is to analyse clinical specimens to test for the presence of microorganisms. The present study aims to apply IMR-MS analysis of headspace gas VC composition to microorganism differentiation by using blood culture broth samples originating from an unselected patient population. In contrast to our previous work on VC based microorganism identification, where a defined group of microorganisms under tight experimental control was analysed, the present work focused on true patient samples that did not undergo any sample preparation or selection prior to VC measurement [19, 20]. To the best of our knowledge the present work represents the first application of VC microorganism identification on clinical samples without sample preparation.

## Results

During the observation period headspace gas was collected from 152 aerobic and 130 anaerobic blood culture broth bottles, which had been identified as positive for microbial growth. Aerobic samples contained 27 Gram-negatives, 106 Gram-positives and 19 fungi whereas in anaerobic samples 37 Gram-negatives, 91 Gram-positives and two fungi were identified. Due to the low number of fungi present in anaerobic samples, they were excluded from further analyses (so that 128 anaerobic samples were left). Among aerobic and anaerobic samples, the most frequently isolated pathogens within the family of Gram-positives were *Staphylococcus epidermidis* (n = 87), *Enterococcus faecium* (n = 23), and *Staphylococcus aureus* (n = 17). Most frequently isolated Gram-negatives were *Escherichia coli* (n = 30) and *Klebsiella pneumoniae* (n = 8). More detailed information about the frequency of isolates in anaerobic samples and their assignment to training and validation set is given in Table 1.

**Table 1:** Anaerobic blood culture broth isolates, set assignment, and results of Gram identification

Gram stain	Microorganism	Isolates (n = 128)		Gram discrimination (n = 42)	
		Training set (n = 86)	Validation set (n = 42)	Identified (n = 35)	Misidentified (n = 7)
Positive	*Enterococcus faecium*	7	3	2	1
	*Listeria monocytogenes*	1	–	–	–
	*Propioninbacterium acnes*	6	1	1	–
	*Streptococcal *sp.				
	*Streptococcus canis*	1	–	–	–
	*S. constellatus*	–	1	1	–
	*S. gallolyticus*	2	3	3	–
	*S. hominis*	1	–	–	–
	*S. mitis*	–	2	2	–
	*S. pneumoniae*	2	–	–	–
	*Staphylococcal* sp.				
	*Staphylococcus aureus*	6	2	2	–
	*S. aureus, methicillin*-*resistant*	4	–	–	–
	*S. capitis*	4	–	–	–
	*S. epidermidis*	24	14	14	–
	*S. haemolyticus*	1	2	1	1
	*S. lugdunensis*	2	2	2	–
Negative	*Enterobacter aerogenes*	2	–	–	–
	*Escherichia coli*	16	7	5	2
	*Citrobacter freundii*	–	1	1	–
	*Klebsiella oxytoca*	1	1	1	–
	*Klebsiella pneumoniae*	3	1	–	1
	*Proteus mirabilis*	–	1	–	1
	*Pseudomonas aeruginosa*	1	–	–	–
	*Salmonella paratyphi*	1	–	–	–
	*Salmonella typhi*	1	1	–	1

**Fig. 1:**
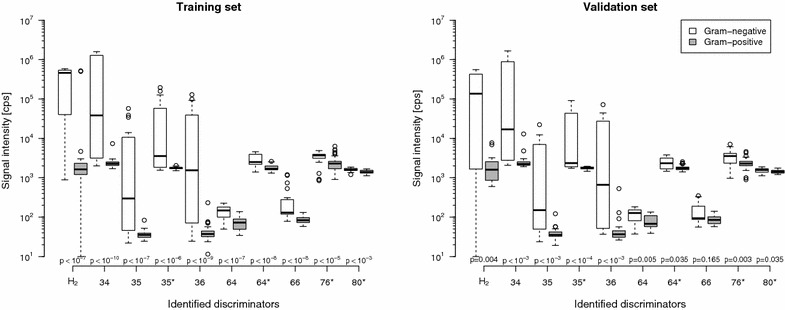
Identified discriminators for differentiation between Gram-negative and Gram-positive bacteria. *Boxplot* of random forest method identified discriminators for the training (*left*) and validation (*right*) set for differentiation into Gram-negative (*white boxplots*) and Gram-positive (*grey boxplots*) bacteria in anaerobic samples. Discriminators are given at their mass to charge ratio (*m/z*) of appearance. H_2_ (*m/z* = 2) was identified using electron impact ionization. Compounds detected at *m/z* = 34–36, 64 and 66 were measured by chemical ionization using mercury as primary ion. Compounds detected at *m/z* = 35, 64, 76 and 80 were measured by chemical ionization using xenon as primary ion. Signal intensity is given in counts per second (cps). *p* values were computed using a Kolmogorov–Smirnov test for testing if distributions were equal in both groups. Due to a previous H_2_ calibration, negative H_2_ values were obtained. Therefore, for the graphical presentation a uniform projection of the H_2_ values into the positive was performed

The headspace VC composition of each microorganism was tested using the measurement results obtained by electron-impact, xenon, and mercury ionization within the above described *m/z* range from 16 to 135. Overall, for each blood culture bottle a complete set of the 198 measurements was available for analysis to discriminate between Gram-negative and -positive. In anaerobic conditions the prediction rules obtained by using the random forest method [22] resulted in CV error rates ranging from 9.1 to 16.4 % for Gram discrimination in the training set (for a range of random forest prediction rules obtained with previous variable selection/without previous variable selection/with previous dimension reduction by partial least squares). Note that a microorganism was classified as Gram-positive if more than 50 % of the trees of the random forest classified the microorganism as Gram-positive. The random forest prediction rule yielding the lowest error rate with 9.1 % was constructed based on 10 *m/z* signals with the highest rankings by random forest’s Gini variable importance measure and with parameter values mtry = 3 and nodesize = 7, where mtry and nodesize denote important technical parameters of the algorithm in the R package randomForest (see Additional file 1 for the performance of the other prediction rules). The subset of *m/z*’s identified in such manner together with the method of ionization and some tentatively assigned compounds are given in Table 2. In Fig. 1, the corresponding signal intensities for the identified *m/z*’s are given.

**Table 2:** Ionization method and masses identified by the random forest method

Ionization	Ion	Identified masses [*m/z*]	Tentative compound
EI-MS	e^−^	2	H_2_
IMR-MS	Hg^+^	34–36, 64, 66	H_2_S (34)^a^
	Xe^+^	35, 64, 76, 80	–

*EI*-*MS* electron impact mass spectrometry; *IMR*-*MS* ion–molecule reaction mass spectrometry

^a^Signalling at *m/z* 36 is 4.20 % of *m/z* 34 signalling which matches exactly the expected value of 4.21 % for the presence of the ^34^S isotope of H_2_S

In the training set estimates for sensitivity (proportion of Gram-positives truly classified as Gram-positive) and specificity (proportion of Gram-negatives truly classified as Gram-negative) were 97.5 and 74.8 %, respectively and a value of 0.93 was obtained for the area under the curve (Table 3). When applying the obtained random forest prediction rule to the validation data set an error rate of 16.7 % with regard to Gram identification was observed. The individually identified microorganisms of samples assigned to the validation data set as well as the results of Gram identification are given in Table 1. The sensitivity of the prediction rule to assign Gram-positive microorganisms correctly was found to be 93.3 % whereas the specificity, which is the correct assignment of Gram-negative microorganisms as Gram-negative, was found to be 58.3 % within the validation data set. The observed area under the curve value of 0.89 was close to the ideal value of 1.00 for both the training and validation data set.

**Table 3:** Accuracy of the random forest prediction rule in training and validation data

	Training set (n = 86)	Validation set (n = 42)
Error rate (%)	9.1	16.7
Sensitivity (%)	97.5	93.3
Specificity (%)	74.8	58.3
AUC	0.93	0.89

*AUC* area under the curve

The analysis of species related differences in signal intensity under anaerobic conditions included solely Gram-positive bacteria as only these appeared in sufficient numbers within our sample. Subsequent analyses aimed to compare the following bacterial species/groups: (i) staphylococci against streptococci, (ii) staphylococci against enterococci, (iii) staphylococci against other Gram-positives, and (iv) enterococci against other Gram-positives. Although several differences appeared between bacterial species/groups these differences were no longer significant after adjusting the results for multiple comparisons using the Bonferroni-Holm method.

In aerobic conditions, the best prediction rule for Gram discrimination yielded a CV error rate of 28.4 % based on the training data and was obtained by using diagonal linear discriminant analysis. This error rate is only marginally smaller than the rate of 30.3 % that would be achieved if all observations were rated as Gram-positive (since Gram-negatives and fungi make up about 30.3 % of the observations in the training data set). Thus, the prediction rule was not considered to be of any practical use and no further analyses were performed and no data are shown.

## Discussion

When analysing the VC headspace composition of patients’ blood culture samples for microorganism detection and identification several problems arise. The main problem one encounters is the lack of standardization procedures. Blood culture samples of patients will largely vary in their composition for technical and patient- specific reasons. Technical reasons include different mixing volumes of blood added to the culture broth sample, variations in temperature exposure and time prior to incubation, exposure of the blood culture bottle content to ambient air via the inserted needle, and insertion of the needle through a liquid disinfectant surface. The latter two of these can lead to headspace contaminations with ambient air or disinfectants. Possible, and even more important, factors to be considered for patient-specific reasons are variations in age, gender, actual disease, pre-existing diseases, organ insufficiencies or failures, applied organ support, medication, nutrition, and inflammation. Thus, the results presented here must be interpreted by taking these factors into account. VC signals detected by the instrument may either originate from the broth medium, the body fluid examined, present microorganisms, physiological- or pathophysiological conditions, use of medications, extracorporeal circuits, or contaminants. The clearest case will be if signals are identified attributable to VCs produced exclusively by microorganisms as for example it is the case for 2-pentylfuran produced by *Aspergillus fumigatus* [23]. Unfortunately, this applies only to a minority of VCs detected as numerous VCs are also produced by human metabolism or are present in nutrition or medication [24, 25]. Therefore, we consider the finding of discriminating bacteria into Gram-negatives and -positives at an error rate of about 17 % still impressive given the numerous factors possibly affecting the sample headspace VC composition that were beyond our control. This can certainly be achieved more accurately by performing a Gram stain, but Gram staining requires the presence of staff, which is usually not present on a 24 h basis. In contrast the method of VC headspace gas analysis can be fully automated and even allows direct messaging of the results via a communication network to the responsible physician independent of routine working hours. Thus the work presented here serves as a “proof of principle” method that can be substituted in the future with a down-scaled MS covering just the absolute necessary number of VC’s required for Gram or species identification. The reasons why the method presented here did not work in aerobic blood culture samples so far remains unclear at the moment. One possible reason might be the ventilation of aerobic blood culture samples against ambient air in order to “oxygenate” the sample; however this assumption is only speculative. In a previous study performed under aerobic conditions using lysogeny broth as culture medium [19], we were able to differentiate between staphylococcal, streptococcal and enterococcal species. Further studies may therefore lead to an adapted methodology that also accomplishes Gram differentiation under aerobic conditions.

A steadily increasing number of publications including our own results report the capability of either direct mass spectrometric as well as GC–MS methods to differentiate between different microorganisms at the species or even strain level [15, 16, 19–21, 26, 27]. A comprehensive review on the subject of mass spectrometric detection of microorganism and mammalian cells was recently published by Chingin et al. [28]. Although promising, it must be noted that so far results were obtained under experimental conditions with stringent control of broth media and bacterial inoculate. Using these precisely controlled conditions the spiking of blood samples with microorganisms allowed for the identification of species specific VCs [14, 29]. Up to now, analyses in blood culture samples and other body fluids of patients with suggested infections were carried out recently by Julák et al. using liquid GC–MS for the identification of volatile and non-volatile fatty acids (VFA/NVFA) that allows identifying anaerobic bacteria [12, 13]. More recently, Schubert et al. applied MALDI-TOF MS for the analysis of clinical blood culture samples, which allowed correct identification of 86.5 % of microorganisms within 25 min of blood culture positive detection [7]. Furthermore, Grundt et al. presented a mass spectrometry based assay for antibiotic susceptibility testing by detecting the β-lactamase hydrolysis product of ampicillin [30].

Our “proof of principle” analysis did not include negative blood culture samples as they became available for analysis only after completion of the routine microbiological investigation period of seven days for blood culture samples. Due to concerns in comparing blood culture samples after such a long period of time with samples detected as “positive” by the BACTEC FX system within an on average considerable shorter time period of 2 days negative samples were disregarded. Nevertheless, further development of the method should certainly include on-line analysis during the whole period of culturing irrespective of the presence of microorganisms to control for matrix interferences and avoid false positive detection of negative samples.

## Conclusions

This study impressively shows that the analysis of VCs in the headspace of anaerobic blood culture samples originating from an unselected patient population has the potential to differentiate between Gram-negative and -positive bacteria despite a large biodiversity present in human blood culture samples due to factors such as age, gender, underlying disease and comorbidity, organ insufficiencies and medication. Our results are promising as they show that the method is technically feasible and allows for the identification of a bacteria Gram typical VC pattern for the first time. Given the ability of the method for automation of blood culture sample analysis it offers the potential to decrease the time to Gram identification, which represents important information to the clinician. Results of VC headspace analysis can be processed without the need of staff presence even outside the usual working hours and provided to the clinician by communication devices to shorten the time required for adaptations of antibiotic treatment in response to microbial test results.

## Methods

The Ethics Committee of the Medical Faculty of the Ludwig-Maximilians-University of Munich waived the need for written informed consent as no patient-related data were collected and stored for the present work.

### Blood cultures and headspace gas sampling

Headspace gas samples of blood culture broths (BD BACTEC Plus Aerobic/F and Anaerobic/F blood culture media; Becton–Dickinson, Heidelberg, Germany) from patients with suspected infection were collected from September to December 2011 when identified as positive for microorganism growth by the BACTEC FX^®^ (BD, New Jersey, USA) blood culture system. Access to the blood culture broth headspace gas was accomplished by introducing a three-way stopcock locked sterile needle through the penetrable membrane. A 25 ml sample of headspace gas was collected under sterile conditions prior to Gram staining of the blood culture broth. The headspace sample was transferred into sterilized evacuated 20 ml glass vials (Macherey–Nagel, Dueren, Germany) and stored at −30 °C until batch analysis. At the time of analysis samples were transferred into a headspace auto sampler (G1888, Agilent technologies, Santa Clara, California, USA) and heated within the integrated oven to a temperature of 65.0 °C. For headspace gas analysis the vials cup was penetrated with a needle and the sample gas was passed through a transfer line heated to 140 °C to the IMR- MS (VF Services GmbH, Absam, Austria) where analyses took place [20].

### Phenotype identification

Gram staining of the blood culture broth sample was carried out immediately following headspace gas collection. Subsequently, depending on Gram staining results, subcultures on Bacteroides Bile Esculin, Candiselect, Chocolate, Columbia blood, MacConkey II, Nagler, Sabouraud, or Vitamin K1 agars were incubated at a temperature of 37 °C. The following day definite isolate identification was performed using MALDI-TOF MS (MALDI Biotyper, BrukerDaltonik GmbH, Bremen, Germany). A Phoenix^®^ (Phoenix, BD Diagnostic Systems, Sparks, MD) automated microbiology system was used for antibiotic susceptibility testing.

### The IMR-MS system

A detailed description of the equipment (Airsense Compact, VF Services GmbH, Absam, Austria) has been published previously [19, 20, 31–33]. In brief, the MS system combines two mass spectrometric techniques, a conventional electron-impact mass spectrometer (EI-MS) for the detection of high e.g. Vol %-concentrations and an IMR-MS. The latter provides a highly sensitive method for on-line and off-line sampling of organic and inorganic compounds and has already been used to determine volatile compounds in exhaled breath [17, 31, 32, 34–36]. The IMR-MS can switch between different positively charged primary ion beams. Available primary ions are generated from krypton, mercury, or xenon gas by electron-impact ionization. These positively charged atomic ions interact with neutral sample gas molecules. Two-body collision processes result in the formation of product ions whenever the ionization potential of the sample molecule is less than the potential energy of the incoming primary ion. Differences in ionization potentials between primary and product ions may result in a bond rupture and hence a lower molecular weight fragment ion. However, in contrast to significant molecule fragmentation observed when applying high-energy electron-impact ionization, the IMR-MS low-energy soft ionization method results in less molecule fragmentation. In our experiments, mercury and xenon ions were used as primary ions. The IMR-MS mass separation is 1 u over the mass range. Between sample measurements the IMR-MS lines where flushed with N_2_ (purity 5.0) to avoid carry over effects.

Based on previous experience [19, 20] with measurements of VCs produced by bacteria the following masses were selected for analysis with a dwell time of 300 ms per mass: using xenon for chemical ionization 16, 17, 20, 21, 26, 27, 29, 30, 32, 33, 35, and 38–122; and mercury for chemical ionization: 17, 19, 28–31, 33–123, and 135. Simultaneous measurements of CO_2_, H_2_, H_2_O, N_2_, and O_2_ concentrations were performed using the EI-MS facility available in the Airsense Compact system.

### Statistical analysis

Prediction rules were derived using the Bioconductor package CMA [37, 38] within the framework of the statistical software R. The Gram stain equivalent (either Gram-positive or Gram-negative) was considered as the response to be predicted by the prediction rule. The CMA package provides automated variable selection, parameter tuning, classifier construction and evaluation for a wide range of standard methods used for the analysis of complex high dimensional data. Several prediction methods, such as boosting, random forest, support vector machines, penalized logistic regression, k nearest neighbours, feed forward and probabilistic neural networks, discriminant analysis, elastic net and lasso-type methods, were applied to obtain prediction rules with the highest possible accuracy. Where applicable, methods were also applied after variable selection and/or dimension reduction of the predictor space. Details are given in Additional file 1.

To assess reliably the performance of the constructed prediction rule and avoid over-optimism a random split validation procedure was adopted [39, 40]. Prior to analysis the data set was randomly split into two non-overlapping subsets (ratio 2:1). Stratified splitting was conducted to preserve the distribution of fungi, Gram-negative and -positive in both subsets. The larger subset (denoted as training set) was used to train and to evaluate a large number of diverse candidate prediction rules and to select the best one. The prediction rule with the lowest error rate was regarded as the best one. The smaller subset (denoted as validation set) was used for the validation of the previously chosen prediction rule in order to obtain a reliable estimate of the expected performance of the prediction rule on future independent data. The assessment of the error of the candidate prediction rules in the training data set was based on fivefold cross validation (CV). Tuning parameters were optimized via nested cross validation, i.e. in each CV-fold a further internal CV is executed in which an optimal value for a tuning parameter is selected out of a range of candidate values. To obtain more stable estimates for the error rate 100 repetitions of fivefold CV were conducted. The analyses were performed separately for aerobic and for anaerobic blood cultures.

Additional analyses were performed to further differentiate between bacterial species within the group of Gram-positives. Prediction rules could not be derived due to the small sample size. Univariate subgroup analyses for detecting significant differences in the distribution of *m/z*’s for bacterial species were conducted using the Kolmogorov–Smirnov test. This nonparametric test was applied for each *m/z* to identify significant differences in distributions for two subgroups. *p* values were adjusted for multiple testing using the Bonferroni-Holm method.

The random forest prediction rule as well as the data and R-code are available under http://www.ibe.med.uni-muenchen.de/organisation/mitarbeiter/020_professuren/boulesteix/bc_headspace/.

## Additional file

10.1186/s40709-016-0040-0 Statistical analysis in anaerobic blood culture samples. In this file the steps for deriving the final random forest prediction rule for the differentiation between Gram-positive and Gram-negative bacteria in anaerobic blood culture samples are described.
