# Supplementary material for: Gram-negative and -positive bacteria differentiation in blood culture samples by headspace volatile compound analysis
Source: J Biol Res (Thessalon). 2016 Mar 12;23:3. doi: 10.1186/s40709-016-0040-0 (PMC4788920; doi:10.1186/s40709-016-0040-0)
Supplement: Supplementary file 1 — 10.1186/s40709-016-0040-0 Statistical analysis in anaerobic blood culture samples. In this file the steps for deriving the final random forest prediction rule for the differentiation between Gram-positive and Gram-negative bacteria in anaerobic blood culture samples are described. [file 40709_2016_40_MOESM1_ESM.pdf]

# Additional file 1

## Statistical analysis in anaerobic blood culture samples

Statistical analysis was performed using the statistical software R (R Core Team; 2013) (version 2.13.1). For the analysis described in this supplement the Bioconductor package CMA (Slawski et al.; 2009) described in Slawski et al. (2008) was used (Biobase version 2.12.2). R-codes for reproducing analysis regarding the anaerobic blood culture samples as well as the data and the random forest prediction rule of the results are available at `http://www.ibe.med.uni-muenchen.de/organisation/mitarbeiter/020_professuren/boulesteix/`.

# 1 Deriving prediction rules on the training data set

This section gives details on classifiers that were fit on the training data set in the anaerobic blood culture samples (and later compared; see Section 2).

*Parameter tuning* was done using internal 3-fold cross-validation. The grids of candidate parameter values were chosen as the default grids in CMA version 1.10.0. Parameters that were not tuned were chosen as the default values in CMA version 1.10.0 if not explicitly specified here.

*Variable selection* was performed before fitting classifiers using the following methods:

- ranking of variables by their $p$-values using a t-test,
- ranking of variables by their $p$-values using Limma (Smyth; 2005) or,
- ranking of variables by their variable importance score via random forest's Gini importance measure (RF VIM).

*Dimension reduction* was performed before fitting classifiers using partial least squares (PLS).

Table S1 gives an overview over the fitted classifiers. All prediction rules listed in Table S1 are made publicly available as Rda-object.

# 2 Comparing the performance of prediction rules

The accuracy of the prediction rules from Section 1 was computed in an unbiased way using 5-fold cross-validation (repeated 100 times), while preserving response class distributions within all folds (stratified sampling). The prediction rules were compared with respect to their cross-validated prediction errors. R-code for comparing the prediction rules can be accessed from the website (see above).
Figures S1 and S2 show the distribution of error rates over the $100 \times 5$ repetitions (prediction rules are in the same order as in Table S1). The minimal error rate is obtained for the random forest prediction rule when using only the 10 highest ranked variables according to the RF VIM. This prediction rule was considered the best.

# 3 Fitting the random forest prediction rule on the whole training data set

The next step was to build the random forest prediction rule with the 10 top ranked variables (by RF VIM) using the whole training data set. This prediction rule might be used for predicting future independent data. First the 10 highest ranked variables by the Gini VIM were identified from the whole training data set. These variables are $H_2$, 34, 35, 35*, 36, 64, 64*, 66, 76*, 80*, whereas the asterisk indicates that compounds were measured by chemical ionization using xenon instead of mercury as primary ion. The tuning parameters (i.e., the number of randomly selected variables at each split and the minimal number of observations in a node) were selected through 3-fold cross-validation performed on the training data set. The optimal value for the number of randomly selected variables at each split (mtry) was 3 and the optimal number for the minimum size of the node (nodesize) was 7. These values were used to build the final random forest prediction rule consisting of 1000 trees based on the whole training set. R-code for fitting the random forest prediction rule on the training data is available at `http://www.ibe.med.uni-muenchen.de/organisation/mitarbeiter/020_professuren/boulesteix/`.

# 4 Validating the random forest prediction rule

The prediction rule derived in the previous section was validated using an independent validation data set that arose from randomly splitting the original data into a training set and the validation set (ratio 2:1). Details on misclassification rate, sensitivity and specificity and the area under the curve are given in the paper. Figure S3 shows the ROC curve for the random forest prediction rule applied to the validation data set (cf. Section 3). R-code for reproducing these results is available at the website. The random forest prediction rule is made available as Rda-object to enable readers to reproduce the analysis and to obtain predictions for the validation data using the 10 top ranked variables $H_2$, 34, 35, 35*, 36, 64, 64*, 66, 76*, 80*.

# References


R Core Team (2013). *R: A Language and Environment for Statistical Computing*, R Foundation for Statistical Computing, Vienna, Austria.
**URL:** *http://www.R-project.org/*

Slawski, M., Boulesteix, A.-L. and Bernau, C. (2009). *CMA: Synthesis of microarray-based classification.* Bioconductor package version 1.10.0.

Slawski, M., Daumer, M. and Boulesteix, A.-L. (2008). CMA–a comprehensive Bioconductor package for supervised classification with high dimensional data, *BMC Bioinformatics* **9**(1): 439.

Smyth, G. K. (2005). Limma: linear models for microarray data, *in* R. Gentleman, V. Carey, W. Huber, R. Irizarry and S. Dudoit (eds), *Bioinformatics and Computational Biology Solutions Using R and Bioconductor*, Statistics for Biology and Health, Springer, pp. 397–420.

| Method | CMA option | Tuned parameters | Variable selection and/or dimension reduction | Parameters differing from default |
|---|---|---|---|---|
| Componentwise boosting with binomial loss function | `compBoostCMA` | no. of boosting steps | - | - |
| Componentwise boosting with exponential loss function | `compBoostCMA` | no. of boosting steps | - | - |
| Componentwise boosting with quadratic loss function | `compBoostCMA` | no. of boosting steps | - | - |
| Tree-based gradient boosting with Bernoulli loss function | `gbmCMA` | no. of trees | - | - |
| Tree-based gradient boosting with exponential loss function | `gbmCMA` | no. of trees | - | - |
| Random forests | `rfCMA` | no. of randomly selected variables at each split; minimal number of observations in a node | - | - |
| Random forests | `pls_rfCMA` | no. of components | PLS | - |
| Random forests with $p \in \{10, 20, \ldots, 100\}$ top variables from variable ranking | `rfCMA` | no. of randomly selected variables at each split; minimal number of observations in a node | RF VIM | - |
| Support vector machines with linear kernel | `svmCMA` | cost parameter | - | - |
| Support vector machines with polynomial kernel | `svmCMA` | cost parameter; degree of the polynomial kernel | - | - |
| Support vector machines with radial kernel | `svmCMA` | cost parameter; width of the radial basis function kernel | - | - |
| L1 penalized logistic regression | `LassoCMA` | penalization parameter | - | - |
| L2 penalized logistic regression | `plrCMA` | penalization parameter | - | - |
| Elastic net penalized logistic regression | `ElasticNetCMA` | penalization parameters for L1 and L2 norm | - | - |
| $k$ nearest neighbors | `knnCMA` | no. of nearest neighbors | - | - |
| $k$ nearest neighbors with $p \in \{10, 20, \ldots, 100\}$ top variables from variable ranking | `knnCMA` | no. of nearest neighbors | $t$-test | - |
| $k$ nearest neighbors with $p \in \{10, 20, \ldots, 100\}$ top variables from variable ranking | `knnCMA` | no. of nearest neighbors | Limma | - |
| Probabilistic $k$ nearest neighbors | `pknnCMA` | no. of nearest neighbors | - | beta = 0.00015 |
| Probabilistic $k$ nearest neighbors with $p \in \{10, 20, \ldots, 100\}$ top variables from variable ranking | `pknnCMA` | no. of nearest neighbors | $t$-test | beta = 0.00015 |
| Probabilistic $k$ nearest neighbors with $p \in \{10, 20, \ldots, 100\}$ top variables from variable ranking | `pknnCMA` | no. of nearest neighbors | Limma | beta = 0.00015 |
| Feed forward neural networks with $p \in \{10, 20, \ldots, 100\}$ top variables from variable ranking | `nnetCMA` | weight decay parameter | $t$-test | - |
| Feed forward neural networks with $p \in \{10, 20, \ldots, 100\}$ top variables from variable ranking | `nnetCMA` | weight decay parameter | Limma | - |

| | | | | |
|---|---|---|---|---|
| Linear discriminant analysis with $p \in \{2, 4, 6, \ldots, 40\}$ top variables from variable ranking | `ldaCMA` | - | $t$-test | - |
| Linear discriminant analysis with $p \in \{2, 4, 6, \ldots, 40\}$ top variables from variable ranking | `ldaCMA` | - | Limma | - |
| Linear discriminant analysis | `pls_ldaCMA` | no. of components | PLS | - |
| Linear discriminant analysis with $p \in \{10, 20, 30, \ldots, 100\}$ top variables from variable ranking | `pls_ldaCMA` | - | $t$-test, PLS | - |
| Linear discriminant analysis with $p \in \{10, 20, 30, \ldots, 100\}$ top variables from variable ranking | `pls_ldaCMA` | - | Limma, PLS | - |
| Fisher's linear discriminant with $p \in \{10, 20, 30, \ldots, 60\}$ top variables from variable ranking | `fdaCMA` | - | $t$-test | - |
| Fisher's linear discriminant with $p \in \{10, 20, 30, \ldots, 60\}$ top variables from variable ranking | `fdaCMA` | - | Limma | - |
| Diagonal linear discriminant analysis | `dldaCMA` | - | - | - |
| Diagonal linear discriminant analysis with $p \in \{5, 10, 15, \ldots, 100\}$ top variables from variable ranking | `dldaCMA` | - | $t$-test | - |
| Diagonal linear discriminant analysis with $p \in \{5, 10, 15, \ldots, 100\}$ top variables from variable ranking | `dldaCMA` | - | Limma | - |
| Quadratic discriminant analysis with $p \in \{2, 4, 6, \ldots, 18\}$ top variables from variable ranking | `qdaCMA` | - | $t$-test | - |
| Quadratic discriminant analysis with $p \in \{2, 4, 6, \ldots, 18\}$ top variables from variable ranking | `qdaCMA` | - | Limma | - |

Table S1: Prediction rules fit on the training data set.

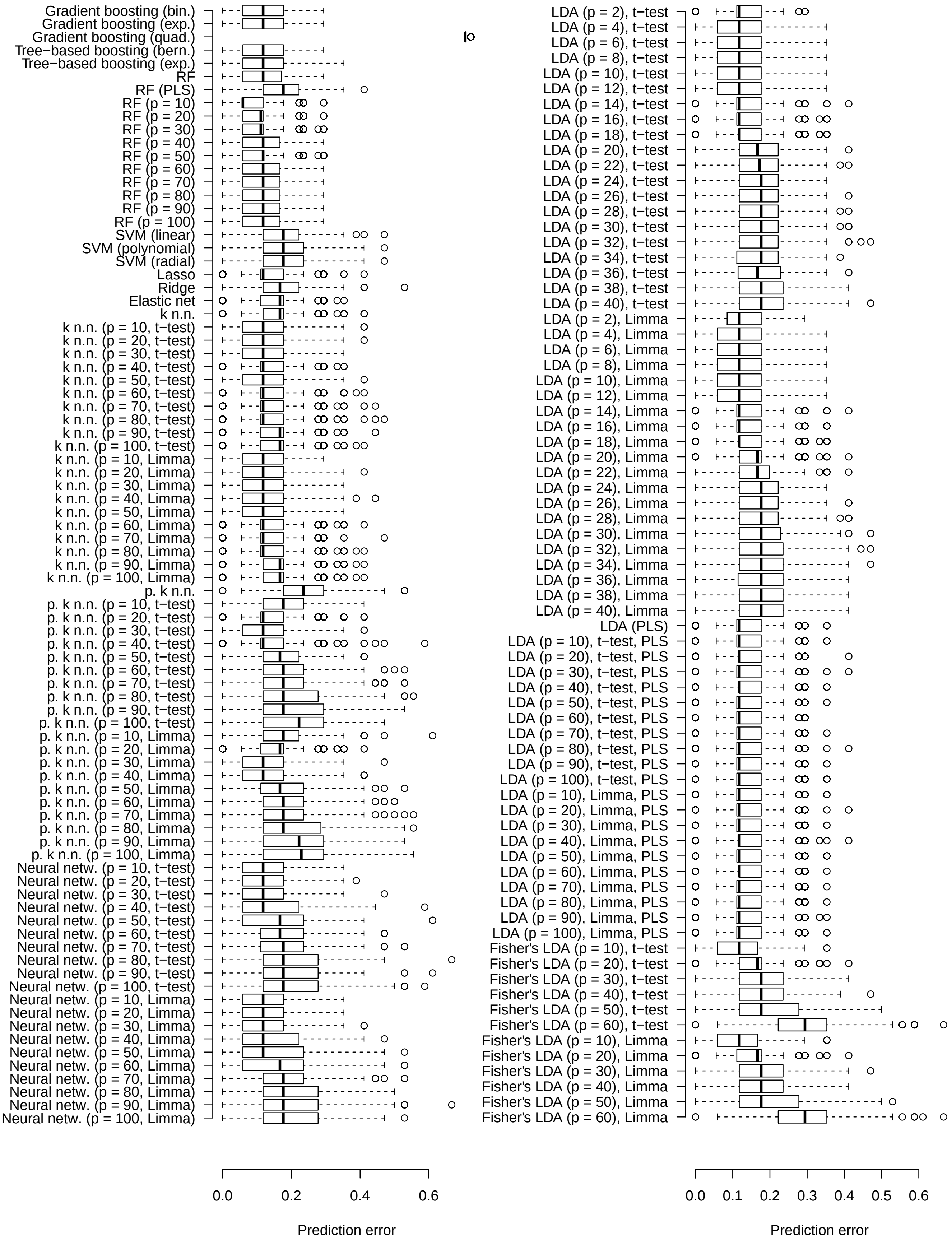


Figure S1: Comparison of prediction rules constructed based on the training data. Parameter $p$ denotes the number of preselected variables. RF: random forests; SVM: support vector machines, k n.n.: $k$ nearest neighbors; p. k n.n.: probabilistic $k$ nearest neighbors; LDA: linear discriminant analysis.

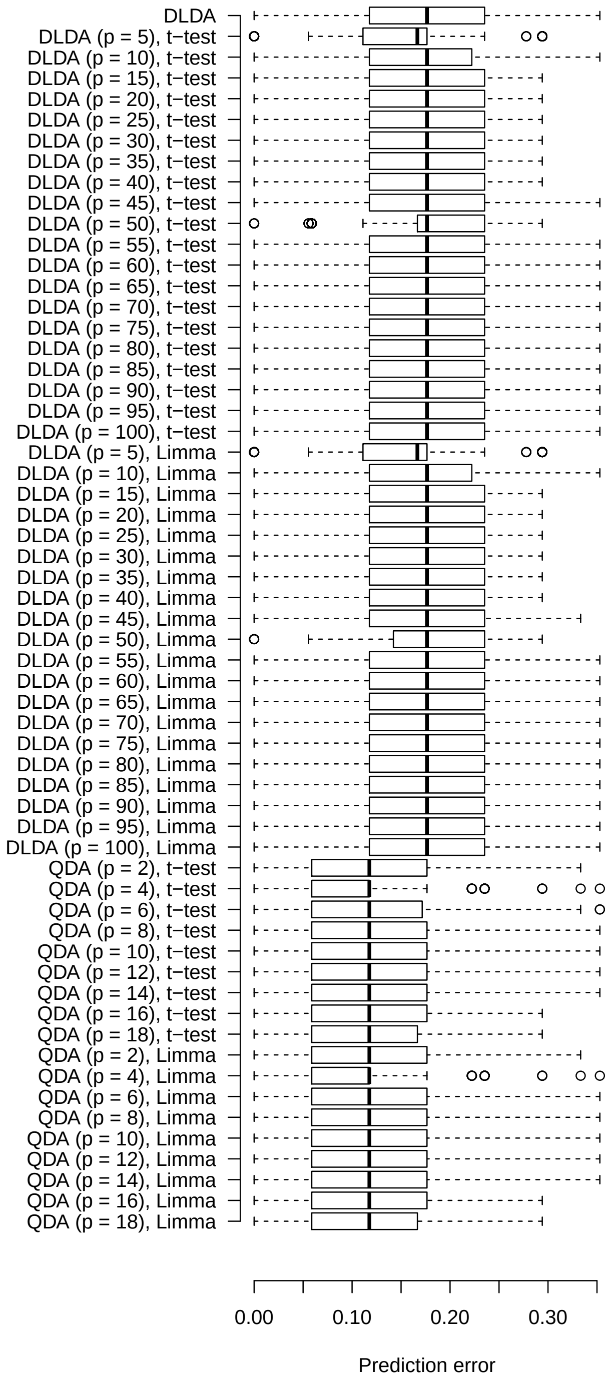


Figure S2: Comparison of prediction rules constructed based on the training data (cont.). Parameter $p$ denotes the number of preselected variables. DLDA: diagonal linear discriminant analysis; QDA: quadratic discriminant analysis.

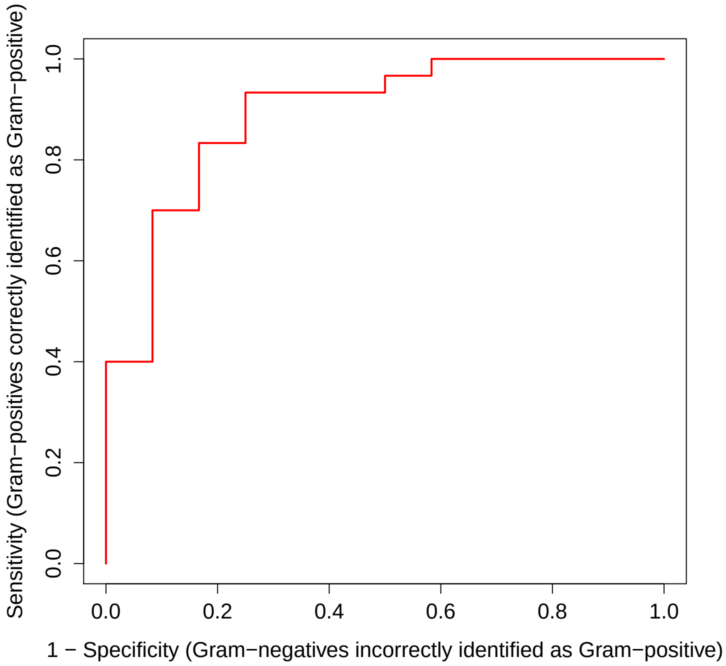


Figure S3: Receiver Operating Characteristic (ROC) curve for the random forest prediction rule evaluated on the validation data set.
